# State and territory immunization program activities and their association with human papillomavirus vaccine initiation in the United States of America: A multilevel approach

**DOI:** 10.1371/journal.pgph.0002852

**Published:** 2024-12-31

**Authors:** Vivian Colón-López, Francisco J. Muñoz-Torres, Erika Escabí Wojna, Idamaris Vega Jimenez, Olga L. Díaz Miranda, Diana T. Medina-Laabes, Katelyn Wells, Ana P. Ortiz, Pamela C. Hull, Erick Suárez

**Affiliations:** 1 Cancer Control and Population Sciences, Comprehensive Cancer Center, University of Puerto Rico, San Juan, Puerto Rico; 2 Association of Immunization Managers, Rockville, MD, United States of America; 3 Department of Behavioral Science, College of Medicine, Markey Cancer Center, University of Kentucky Lexington, Lexington, KY, United States of America; National University of Singapore, SINGAPORE

## Abstract

This study evaluates the association between immunization program (IP) activities aimed at increasing HPV vaccination among adolescents and their impact on initiation rates. Our data sources are: (i) 2016 AIM Annual Survey and (ii) 2019 National Immunization Survey–Teen. We estimated the prevalence of HPV vaccine initiation using a multilevel Poisson model, combining state-level IP data and individual characteristics of adolescents. We calculated the prevalence ratio (PR) of HPV initiation among adolescents to compare the effects of IP activities, adjusting for state of residence, age, sex, maternal education, and ethnicity. A total of 17,390 teens aged 13 and 17 were evaluated. States with publicly available school-based adolescent coverage rates and/or exemptions (PR^w, activity D^_adjusted_: 1.08, 95% CI: 1.02, 1.14), and those that expanded the number of pharmacies entering HPV vaccination data (PR^w, activity N^_adjusted;_ 1.06, 95% CI: 1.02, 1.10) in Immunization Information Systems (IIS), had higher HPV vaccine initiation rates compared to states that did not implement these strategies. When stratifying, these findings were present in the younger group (13–15 years, PR^w, activity D^_adjusted_: 1.10, 95% CI: 1.01, 1.18; PR^w. activity N^_adjusted_: 1.10, 95% CI: 1.05, 1.16), but not in the older group (16–17 years, PR^w, activity D^_adjusted_: 1.05, 95% CI: 0.95, 1.15; PR^w. activity N^_adjusted_: 1.00, 95% CI: 0.94, 1.06). States that expanded the number of school-located programs entering HPV vaccine records in IIS (PR^w, activity E^_adjusted_: 1.08, 95% CI: 1.01, 1.15) had higher vaccine initiation prevalence in the younger group but not in the older group. Limitations include a lack of operational definitions for IP activities, potential biases in the NIS-Teen survey, and reliance on provider-reported HPV vaccination. Nonetheless, these results highlight immunization activities that support national efforts to increase HPV vaccine uptake and inform public health programs on effective HPV vaccine promotion.

## Introduction

The efficacy of HPV vaccines has been demonstrated in multiple clinical trials, which have shown an overall reduction of HPV-related diseases through individual vaccination and herd immunity [1]. Despite the efficacy of the HPV vaccine in preventing HPV infection, genital warts, and high-grade precancerous cell changes in the cervix [2], the United States of America (US) has not achieved optimal completion rates according to the objectives delineated by *Healthy People 2020 and 2030* [3, 4]. In 2020, 75.1% of adolescents aged 13–17 had received ≥1 dose of the HPV vaccine, and 58.6% were considered HPV Up-to-Date (UTD) [5]. Studies have also documented that disparities remain, meaning that all adolescents are not equally protected against HPV vaccine-preventable diseases [5, 6].

Achieving optimal HPV vaccination uptake is a public health priority for state, local, and territory immunization program (IP) managers [7] promoting HPV vaccination through activities and efforts to increase community engagement across states and territories. The 64 IP managers lead state, local, and territorial IPs, directing public health efforts to keep children and adults vaccinated and protected against vaccine-preventable diseases [8]. The Association of Immunization Managers (AIM) was created in 1999 to support immunization managers in effectively preventing and controlling vaccine-preventable diseases and improving immunization coverage in the USA and its territories [8].

When pursuing the goal of increasing HPV vaccination in the US, it is important to consider the factors that interplay and influence HPV vaccination rates. These factors span several levels: individual (characteristics of an individual), interpersonal (networks and social support systems), institutional/organizational (entities with rules or regulations that affect immunization services), community (relationships among organizations, institutions, and informational networks), and policy (local, state, national and global laws, and policies) [9–12]. Most strategies have targeted the individual level [13]. For example, several communities and specific subgroups have implemented health provider reminders (from doctors, nurses, pharmacists, and community health workers) and community engagement strategies through social media messages, billboards, radio, or face-to-face meetings with parents and their adolescents [14]. However, few scientific studies have examined how factors at higher socioecological framework levels (e.g., institutional-, community-, or policy-level factors) may influence HPV vaccination rates [15, 16]. It is important to account for the hierarchical and interconnected nature of the factors influencing vaccination decisions. A more comprehensive approach is necessary to consider factors at multiple levels, encompassing state, community, and policy factors.

The specific impact of state-level initiatives conducted by Departments of Health remains understudied. Moreover, data are also scarce in understanding the synergistic importance of community alliances and coalitions in improving HPV vaccination rates nationally. To address these gaps, this study evaluated the effectiveness of IP activities conducted by state immunization programs in increasing adolescent HPV vaccine initiation across the US in 2019. By integrating these perspectives, our study contributes uniquely to the current understanding, guiding the development of more effective, contextually tailored public health interventions.

## Materials and methods

### Participants and data sources

This study did not have an a priori protocol, and thus, no formal registration was conducted with any study registry. As no a priori protocol was established, there were no post hoc changes to report.

This study combined databases from two different sources. First, to compute the HPV initiation prevalence, we used data from the National Immunization Survey for Teens (NIS-Teen) of 2019. The NIS-Teen is an observational, random-digit-dialing telephone survey of parents or guardians of teens ages 13 to 17 years, followed by a mailed survey to the teens’ immunization providers to collect and monitor vaccination coverage. This survey includes information regarding routine adolescent vaccines, such as tetanus, diphtheria, acellular pertussis, meningococcal conjugate, HPV, and influenza vaccines [14]. This dataset provides provider data, confirmed by the health providers in the second phase of the survey, which helps avoid bias in the data collection process [17]. The NIS-Teen 2019 data represents a national probability sample of teens living in the USA and some of its territories, where sampling weights are provided for the teen participants and the healthcare providers [18]. For this study, we focused on provider-reported HPV vaccination history among teens (*provider data)*. Thus, adolescents included in the analysis had to have adequate provider data [17]. The target sample size of completed interviews in each estimation area is designed to achieve an approximately equal coefficient of variation of 6.5% for an estimator of vaccination coverage derived from provider-reported vaccination histories [18]. Detailed methodology and weighting procedures are described in the NIS-Teen User’s Guide for the 2019 Public-Use Data File [18].

The NIS-Teen survey in 2019 gathered observations of a total of 43,503 teens, with an interview completion rate of 71.5%, corresponding to the parents or caregivers who responded to the interview. From this total, only 44% of the teens had adequate provider data [18] based on their vaccination records for a total of 18,722 teens residing in any of the 61 geographic strata included in the 2019 NIS-Teen Survey; 7 local areas (Bexar County, TX; City of Chicago, IL; City of Houston, TX; New York City, NY; Philadelphia County, PA; Dallas County, TX; El Paso County, TX), the 50 states, District of Columbia, and three territories (the U.S. Virgin Islands, Guam, and Puerto Rico) [18].

The AIM Annual Survey explores state, local, and territorial IP policies, infrastructure, IP activities, priorities, and the impact of funding changes (both federal and state) on immunization programs of all 64 member states and territories [19]. We used information from the AIM Annual Survey conducted in 2016 to assess 14 immunization activities that promote HPV vaccination among adolescents at the state level. For this study, data from 49 states and the territory of Puerto Rico were considered for analysis. The AIM survey data gathered was from 3 years before the outcome of interest (NIS-Teen 2019) to provide a period to assess the effectiveness of IP activities conducted by state immunization programs. One state did not respond to whether it had carried out three immunization activities listed in the AIM Annual Survey. As a result, we did not include 602 observations, representing only 3.3% of the overall NIS-Teens data. To assess any selection bias, we compared observations with complete data to the previously described incomplete observations. They had similar percentages of teens aged 13–15 years (62.2% vs. 63.5%), males (47.6% vs 45.5%), mothers with higher than high school education (76.2% vs. 71.4%), and Hispanics (19.4% vs. 27.2%). Due to this similarity, we pursued the analysis for this study with 17,390 observations with complete data.

## Ethics statement

The study was approved by the Institutional Review Board of the University of Puerto Rico Comprehensive Cancer Center (protocol number 2023-04-98). Formal consent was not applicable; unidentified data was used for secondary analysis.

To ensure privacy and confidentiality, all data used in this study were de-identified prior to analysis. Personally identifiable information was removed or replaced with anonymous identifiers. Access to the dataset was restricted to authorized personnel, and stringent data protection protocols were implemented to prevent unauthorized access or disclosure. This study complied with relevant regulations and guidelines for data privacy and confidentiality, including the Institutional Review Board (IRB) approval.

### Individual and state-level variables

#### Main outcome: HPV vaccine initiation at an individual level

HPV vaccine initiation data were derived from the National Immunization Survey for Teens (NIS-Teen) of 2019. The prevalence of HPV vaccine initiation was computed based on provider-reported vaccination histories among teens, with initiation defined as receiving at least one dose. This provider-reported individual-level variable indicates the proportion of adolescents with at least one dose of the HPV vaccine administered among teens in the National Immunization Survey for Teens (NIS-Teen) of 2019 [20].

#### Main predictor variables: Immunization program activities at a state level

IP activities were obtained from the 2016 Association of Immunization Managers (AIM) Annual Survey. These activities are described in Table 1. IPs rated their state’s level of engagement for each activity on a scale from 1 to 5 as follows: (1) Did not engage/not a priority; (2) Did not engage but would like to if resources were available; (3) Had some engagement in activity but could not expand because of limited resources; (4) Had some engagement which was all that was needed; and (5) High level of engagement because this is part of our program’s core activities. Responses for each activity were dichotomized into two possible categories: **0** or **1**. Where **0** represents those IPs that did not engage with the activity (regardless of their reason), and **1** represents the programs that engaged in the activity (without distinguishing if the engagement was some or high). Therefore, categories 1 and 2 were collapsed into one new category (**0**), and categories 3, 4 and 5 were collapsed into another (**1**).

**Table 1:** Immunization program activities based on the AIM annual survey conducted in 2016.

Item	States Performing n (%)
A. Expand the number of OB/GYN providers enrolled in the Vaccine for Children (VFC) program.	24 (49.0)
B. Assess adolescent coverage during Assessment, Feedback, Incentives, and eXchange (AFIX) visits.	44 (89.8)
C. Provide adolescent coverage rates and/or exemptions reports to the Department of Education by school.	19 (38.8)
D. Make school-based adolescent coverage rates and/or exemptions available to the public (*e*.*g*., a dashboard on a website with county level coverage and exemption rates).	20 (40.8)
E. Expand the number of school-located programs entering HPV in IIS.	14 (28.6)
F. Implement IIS reminder/recall system through a centralized approach.	19 (38.8)
G. Implement IIS reminder/ recall by providing support to providers	30 (61.2)
H. Provide CDC ‘You are the Key to Cancer Prevention’ resources to providers.	42 (85.7)
I. Offer provider Continuing Medical/Nursing Education (CME/CNE) programs about the HPV vaccine.	30 (61.2)
J. Conduct educational webinars targeting providers that focus on the HPV vaccine.	31 (63.3)
K. Conduct education/outreach to increase the public’s knowledge regarding HPV vaccination.	38 (77.6)
L. Develop HPV vaccine promotion messages using social media.	31 (63.3)
M. Expand the number of pharmacists enrolled in the VFC program.	13 (26.5)
N. Expand the number of pharmacies entering HPV vaccination data in IIS.	27 (55.1)

#### Other covariates

To assess the effect of potential confounders in the relationship between IP activities and HPV vaccine initiation, the Poisson regression model was fitted for several individual-level demographic covariates. Based on the literature review, we considered the following variables as potential confounders in this model: sex (male or female), adolescent age (13 to 17 years), race and ethnicity (Hispanic, non-Hispanic White, non-Hispanic Black, or non-Hispanic Multiracial), and education attained by the mother (equal or less than high school or more than high school) [21–24]. By including these covariates in the model, we aimed to estimate the adjusted prevalence ratio of the association between each IP activity and the HPV vaccine initiation prevalence rate, according to the NIS-Teen 2019 survey.

#### Multilevel modeling

The multilevel modeling approach was used to reach our aim due to the complexity of factors influencing HPV vaccine initiation at different levels and contexts, such as the individual characteristics of every teen, provider, and state. Therefore, a method capable of simultaneously examining these multifaceted influences is needed. Vaccination decisions are not made in isolation; they are embedded within a hierarchical structure of factors, with individuals nested within communities and communities within states. Multilevel modeling enhances statistical efficiency by appropriately accounting for the nested nature of the data, leading to more precise parameter estimates and reducing the risk of biased results. This approach facilitates a comprehensive understanding of the mechanisms underlying HPV vaccine initiation by examining factors at multiple levels concurrently, enabling the identification of individual, and state-level determinants of vaccination behavior and informing the development of targeted interventions. By simultaneously considering factors at various levels, this methodological approach offers a robust framework for studying the association between IP activities and vaccination behavior, ultimately contributing to a nuanced understanding of how to promote HPV vaccine uptake rates effectively.

Therefore, to combine our data with different levels of information (individual and state characteristics), we used a mixed regression model with random intercept and exchangeable correlation to assess the effects of state-level factors on HPV vaccine initiation prevalence, controlling for different confounders, as described previously.

Initially, to describe the pattern of HPV vaccination in the USA using data at the state and territorial level, the vaccine initiation prevalence rate for HPV among teens was estimated overall and by specific demographic characteristics measured at the individual level, as follows:

$$HPV\;initiation\;prevalence=\frac{Number\;of\;teens\;interviewed\;that\;initiated\;the\;HPV\;vaccine}{Total\;of\;teens\;interviewed}$$

Afterward, we fitted the HPV initiation prevalence utilizing a mixed-effects Poisson regression model with random intercept [25], as follows:

$$\mu_{ik}=T_{k}*e^{\beta_{0k}+\sum A_{i}+\sum \theta_{j}C_{j}}$$

Where:

*μ*_*ik*_ expected number of teens that initiated the HPV vaccine in a state with the *i*-IP activity in the *k*-state

*T*_*k*_ indicates the total number of teens interviewed

A_i_ indicates the effect in the number of teens that initiated the HPV vaccine in a state with the *i*-IP activity.

C_j_ indicates the *j*-the potential confounder

*θ*_*j*_ indicates the effect in the number of teens that initiated the HPV vaccine in the *j*-confounder

$$\beta_{ok}=\gamma_{00}+u_{ok}$$

*γ*_00_ indicates the average in the number of teens that initiated the HPV vaccine at the state level (constant term)

$u_{0k}∼N(0,\sigma_{uo}^{2})$ indicates the difference between *γ*_00_ and the number of teens that initiated the HPV vaccine at state level. We assume that this difference follows a Normal distribution with mean 0 and variance $\sigma_{u0}^{2}$.

To accomplish the assumption of Poisson distribution in this model, we assessed overdispersion by comparing the deviance of the model to its degrees of freedom, given that the IP activities applied to a grouped number of adolescents. The estimated ratio of deviance to degrees of freedom was 0.90, which is close to 1, indicating that overdispersion is unlikely. Additionally, we performed a chi-square test to compare the two values, and the resulting p-value was 0.53. This suggests that there is no significant overdispersion, allowing us to confidently use the Poisson regression model. To account for the hierarchical structure of the data, with individuals nested within states, we conducted a multilevel analysis with the following levels (i) individual-level variables used to identify characteristics of each teen and (ii) state-level variables representing IP activities. Therefore, the mixed Poisson regression model was employed to estimate the effects of IP activities on HPV vaccine initiation prevalence rate while adjusting for individual-level characteristics and accounting for clustering of individuals within states. To perform our parameters estimation, we normalized the weighted factors provided by NIS-Teen 2019 to account for the sampling design effect as follows:

$$\frac{Original\;weight\;}{Average\;weight}$$

Based on this model, we estimated the magnitude of the association between these activities and HPV vaccine initiation using the Prevalence Ratio (PR) with 95% confidence intervals, considered statistically significant if p < 0.05. An assessment of interaction terms between all the predictors in the Poisson model was also performed using the likelihood ratio test; however, we did not find any significant interaction terms (p > 0.05). We also explored whether the age group modifies the statistical relationship between the specific activity and the prevalence of HPV vaccine initiation.

In our multilevel Poisson regression analysis, we explicitly accounted for the extent of state-level variation in HPV vaccine initiation rates, and we considered the concept of residual variance at the state level as a measure of state-level variation. Additionally, the significance of the random effects term in the multilevel Poisson regression model was assessed using likelihood ratio tests, indicating significant variation in HPV vaccine initiation rates across states after accounting for the effects of IP activities and individual-level covariates. All the statistical analyses were performed using Stata version 18 (US: StataCorp LLC) using the *mepoisson* with an exchangeable correlation option.

## Results

### Teens in NIS Data 2019 by sociodemographic characteristics residents of USA states and Puerto Rico

Table 2 shows the number of teens in NIS 2019 data by sociodemographic characteristics. The overall number of parents interviewed was 17,390 within the different USA states and Puerto Rico. Around 52.4% were males, and 62.2% were 13 to 15 years old. The educational attainment of most of the mothers of these teens (76.2%) was more than high school. Approximately two-thirds of the interviewees (63.9%) were self-identified as Non-Hispanic White. The overall HPV initiation unweighted prevalence was 71.2%.

**Table 2:** Number of teens in NIS data 2019 by sociodemographic characteristics residents of USA states and Puerto Rico.

Characteristic	Interviewed (n)	% (95% CI)
**Total**	**17,390**	**100**
**Child’s Sex**		
Male	9,117	52.4 (51.7, 23.2)
Female	8,273	47.6 (46.7, 48.3)
**Age** (years)		
13–15	10,821	62.2 (61.5, 62.9)
16–17	6,569	37.8 (37.1, 38.5)
**Education attainment of the mother**		
High School or Less	4,137	23.8 (2.1, 24.4)
More than High school	13,253	76.2 (75.6, 76.8)
**Ethnicity**		
Hispanic	3,374	19.4 (18.8, 20.0)
Non-Hispanic White	11,118	63.9 (63.2, 64.6)
Non-Hispanic Black	1,199	6.9 (6.5, 7.3)
Non-Hispanic Multiracial	1,699	9.8 (9.3, 10.2)
**HPV initiation**		
Yes	12,385	71.2 (70.5, 71.9)
No	5,005	28.8 (28.1, 29.5)

## Immunization program activities

Activities with a higher association with HPV initiation prevalence at the state level are described in Table 3. HPV vaccine initiation prevalence among states that (**D**) made school-based adolescent coverage rates and/or exemptions available to the public was 8% higher (PR^w, activity D^_adjusted_: 1.08, 95% CI: 1.02, 1.14) than states that did not, and (**N**) states that expanded the number of pharmacies entering HPV vaccination data in the Immunization Information System (IIS) showed 6% higher prevalence (PR^w, activity N^_adjusted_: 1.06, 95% CI: 1.02, 1.10) than those states that did not expand them.

**Table 3:** Magnitude of association between IP activities and HPV vaccine initiation.

Activities	Performed at the state level?	HPV vaccine initiation? (n = 17, 390)		Weighted Prevalence Ratio (PR^W)^ (95% CI)^a^	
		Yes	No	Model 1	Model 2
A. Expand the number of OB/GYN providers enrolled in the VFC program	**Yes**	6,884	5,501	0.99 [0.94, 1.03]	0.99 [0.94, 1.03]
	**No**	2,849	2,156	1.00	1.00
B. Assess adolescent coverage during AFIX visits	**Yes**	11,349	1,036	0.95 [0.89, 1.03]	0.97 [0.90, 1.04]
	**No**	4,498	507	1.00	1.00
C. Provide adolescent coverage rates and/or exemptions reports to the Department of Education by school	**Yes**	5,414	6,971	1.03 [0.98, 1.09]	1.02 [0.97, 1.08]
	**No**	2,083	2,922	1.00	1.00
D. Make school-based adolescent coverage rates and/or exemptions available to the public	**Yes**	5,958	6,427	1.06 [1.00, 1.12]*	1.08 [1.02, 1.14]*
	**No**	2,306	2,699	1.00	1.00
E. Expand the number of school-located programs entering HPV in IIS	**Yes**	3,310	9,075	1.03 [0.98, 1.09]	1.04 [0.99, 1.10]
	**No**	1,221	3,784	1.00	1.00
F. Implement IIS reminder/ recall through a centralized approach	**Yes **	4,533	7,852	1.03 [0.98, 1.08]	1.04 [0.99, 1.10]
	**No **	1,652	3,353	1.00	1.00
G. Implement IIS reminder/ recall by providing support to providers	**Yes**	7,029	5,356	1.02 [0.97, 1.07]	1.02 [0.97, 1.07]
	**No**	2,801	2,204	1.00	1.00
H. Provide CDC ‘You are the Key to Cancer Prevention’ resources to providers	**Yes**	10,782	1,603	0.97 [0.90, 1.04]	0.98 [0.92, 1.06]
	**No**	4,388	617	1.00	1.00
I. Offer provider CME/CNE programs about the HPV vaccine	**Yes **	8,054	4,331	0.97 [0.92, 1.03]	0.96 [0.91, 1.02]
	**No **	3,262	1,743	1.00	1.00
J. Conduct provider educational webinars that focus on HPV	**Yes **	8,487	3,898	0.98 [0.93, 1.04]	0.98 [0.92, 1.03]
	**No **	3,319	1,686	1.00	1.00
K. Conduct education/outreach to increase the public’s knowledge regarding HPV vaccination	**Yes**	10,100	2,285	1.03 [0.96, 1.11]	1.03 [0.96, 1.11]
	**No**	3,916	1,089	1.00	1.00
L. HPV messaging using social media	**Yes**	7,923	4,462	0.99 [0.93, 1.05]	0.97 [0.92, 1.03]
	**No**	3,100	1,905	1.00	1.00
M. Expand the number of pharmacists enrolled in the VFC program	**Yes**	3,997	8,388	0.96 [0.91, 1.02]	0.97 [0.91, 1.02]
	**No**	1,593	3,412	1.00	1.00
N. Expand the number of pharmacies entering HPV vaccination data in IIS	**Yes**	5,910	6,475	**1.05 [1.01, 1.09]**	1.06 [1.02, 1.10]*
	**No**	2,307	2,698	1.00	1.00
${\hat{Var}}_{Between\;state}$				1.25*e*−30	2.44*e*−31

*p-value < 0.05

**a** Weighted analysis was performed in this model after normalizing the individual weights provided by NIS- Teen 2019

**Model1** Adjusted for all the IP activities, controlling the effect of state of residence.

**Model2** Additionally, adjusting for age, sex, educational attainment of the mother, and ethnicity.

The age-stratified analysis (Table 4), in which observations were stratified into two age groups, individuals aged 13–15 years and those aged 16–17 years, reveals significant associations (p<0.05). Among teens aged 13–15 years, states that **(D)** made school-based adolescent coverage rates and/or exemptions available to the public exhibited a 10% higher prevalence of vaccine initiation (PR^w, activity D^_adjusted_: 1.10, 95% CI: 1.01, 1.18), states that **(E)** expanded the number of school-located programs integrating HPV into the Immunization Information System showed an 8% higher prevalence of vaccine initiation (PR^w, activity E^_adjusted_: 1.08, 95% CI: 1.01, 1.15), and states that **(N)** increased the number of pharmacies reporting HPV vaccination data in the Immunization Information System showed a 10% higher initiation prevalence (PR^w, activity N^_adjusted_: 1.10, 95% CI: 1.05, 1.16) compared to states that did not undertake these activities. Among teens aged 16–17, states that **(M)** expanded the number of pharmacists enrolled in the VFC program showed a lower prevalence of vaccine initiation (PR^w, activity M^_adjusted_: 0.89, 95% CI: 0.81, 0.97) compared to states that did not.

**Table 4:** Age subgroup analysis for the association between IP activities and HPV vaccine initiation.

Activities	Performed at the state level?	HPV vaccine initiation in		PR^W^	HPV vaccine initiation in		PR^W^
		*13–15 years*		(95% CI)^a^	*16–17 years*		(95% CI)^a^
					*n = 6*,*569*		
		*n = 10*,*821*					
		Yes	No	Adjusted^b^	Yes	No	Adjusted^b^
A. Expand the number of OB/GYN providers enrolled in the VFC program							
	**Yes **	4,240	3,358	0.98 [0.92, 1.04]	2,644	2,143	1.00 [0.93, 1.08]
	**No **	1,861	1,362	1.00	988	794	1.00
B. Assess adolescent coverage during AFIX visits							
	**Yes **	6,978	620	0.91 [0.83, 1.00]	4,371	416	1.06 [0.94, 1.19]
	**No **	2,909	314	1.00	1,589	193	1.00
C. Provide adolescent coverage rates and/or exemptions reports to the Department of Education by school							
	**Yes **	3,360	4,238	0.99 [0.92, 1.06]	2,054	2,733	1.08 [0.99, 1.17]
	**No **	1,349	1,874	1.00	734	1,048	1.00
D. Make school-based adolescent coverage rates and/or exemptions available to the public							
	**Yes **	3,661	3,937	1.10 [1.01, 1.18]*	2,297	2,490	1.05 [0.95, 1.15]
	**No **	1,467	1,756	1.00	839	943	1.00
E. Expand the number of school-located programs entering HPV in IIS							
	**Yes **	2,051	5,547	1.08 [1.01, 1.15]*	1,259	3,528	0.98 [0.90, 1.07]
	**No **	752	2,471	1.00	469	1,313	1.00
F. Implement IIS reminder/ recall through a centralized approach							
	**Yes **	2,770	4,828	1.04 [0.98, 1.11]	1,763	3,024	1.05 [0.97, 1.13]
	**No **	1,076	2,147	1.00	576	1,206	1.00
G. Implement IIS reminder/ recall by providing support to providers							
	**Yes **	4,311	3,287	1.00 [0.94, 1.07]	2,718	2,069	1.04 [0.96, 1.13]
	**No **	1,802	1,421	1.00	999	783	1.00
H. Provide CDC ‘You are the Key to Cancer Prevention’ resources to providers							
	**Yes **	6,659	939	1.02 [0.92, 1.12]	4,123	664	0.95 [0.85, 1.06]
	**No **	2,825	398	1.00	1,563	219	1.00
I. Offer provider CME/CNE programs about the HPV vaccine							
	**Yes **	4,941	2,657	0.99 [0.93, 1.06]	3,113	1,674	0.93 [0.85, 1.01]
	**No **	2,069	1,154	1.00	1,193	589	1.00
J. Conduct provider educational webinars that focus on HPV							
	**Yes **	5,292	2,306	0.97 [0.91, 1.04]	3,195	1,592	0.98 [0.90, 1.07]
	**No **	2,138	1,085	1.00	1,181	601	1.00
K. Conduct education/outreach to increase the public’s knowledge regarding HPV vaccination							
	**Yes **	6,196	1,402	1.01 [0.92, 1.11]	3,904	883	1.05 [0.94, 1.18]
	**No **	2,538	685	1.00	1,378	404	1.00
L. HPV messaging using social media							
	**Yes **	4,879	2,719	0.95 [0.88, 1.03]	3,044	1,743	1.00 [0.91, 1.10]
	**No **	1,998	1,225	1.00	1,102	680	1.00
M. Expand the number of pharmacists enrolled in the VFC program							
	**Yes **	2,506	5,092	1.02 [0.94, 1.10]	1,491	3,296	0.89 [0.81, 0.97]*
	**No **	992	2,231	1.00	601	1,181	1.00
N. Expand the number of pharmacies entering HPV vaccination data in IIS				** **			
	**Yes **	3,690	3,908	1.10 [1.05, 1.16]*	2,220	2,567	1.00 [0.94, 1.06]
	**No **	1,478	1,745	1.00	829	953	1.00
${\hat{Var}}_{Between\;states}$		3.82*e*−34			1.16*e*−34		

*p-value < 0.05

**a** Weighted analysis was performed in this model after normalizing the individual weights provided by NIS- Teen 2019

**b** Adjusted for all IP activities, sex, educational attainment of the mother, and ethnicity. Additionally, it controls the effect of state of residence.

The between-group variance provides key insights into the extent of state-level impact on HPV vaccine initiation. A slight between-group variance suggests that the differences in HPV vaccine initiation rates between states are minimal. This reinforces that state-level factors, other than IP activities, may not significantly contribute to the observed variation in HPV vaccine initiation rates across states.

The most frequently carried out activities were: (**N**) expanding the number of pharmacies entering HPV vaccination data in IIS, carried out in 27 states, (**D**) making school-based adolescent coverage rates and/or exemptions available to the public, carried out in 20 states, and (**E**) expanding the number of school-located programs entering HPV in IIS, carried out in 14 states.

## Discussion

### Overall findings

This study evaluates the influence that state-level community-based IP activities have on HPV vaccine initiation among adolescents in the USA and its territories after adjusting for individual-level factors. Our analysis suggests that specific community IP strategies can significantly influence the uptake of HPV vaccine initiation. Previous studies centering on vaccination at a state level in the USA have indicated that vaccination rates vary between states and that individual characteristics affect vaccination coverage [26, 27].

In a study conducted to evaluate the effect of state-level characteristics on adult vaccination coverage in the USA, all estimated MORs (median odds ratios) were > 1, indicating that state of residence may affect an individual’s likelihood of vaccination [28]. Based on our findings, states that provide school-based adolescent coverage rates and/or exemptions available to the public presented significantly higher initiation rates (activity D). This result suggests that contextualized information might help to increase HPV vaccine uptake [29]. A study [30] found that 44% of hesitant participants indicated a lack of available information about the HPV vaccine. This highlighted the public’s need for transparency and reliable information about HPV vaccination rates, translating into greater public confidence in the HPV vaccine.

Our findings also suggest that states expanding the number of school-located programs entering HPV in IIS (*activity E*) and states increasing the number of pharmacies entering HPV vaccination data in IIS showed higher initiation rates (*activity n*). These findings are consistent with previous studies carried out in the USA that have found that increased vaccine availability, such as school-based programs, is associated with HPV vaccine uptake or completion [31–33].

On the other hand, our findings also suggest that states that expanded the number of pharmacists enrolled in the VFC program had lower HPV vaccine uptake among older teenagers (*activity M*).

### Implications for research

In addition to the previously proposed recommendations for quantifying and enhancing the understanding of nationwide HPV vaccination efforts, future studies should explore the uptake of other adolescent vaccines and assess how state immunization programs (IPs) have been impacted by the COVID-19 pandemic. It is also essential to analyze trends in HPV initiation rates over time by state, considering the implementation dates of specific initiatives. By tracking these initiation rates and correlating them with the timing of particular IP interventions, researchers can evaluate the effectiveness of these activities in influencing vaccine uptake. Furthermore, specific state policies regarding pharmacy and school-based vaccination programs, their distribution frequency, and their relationship to HPV vaccine uptake should be examined.

The associations observed might be due to a ‘more is more’ approach in IP strategies to increase HPV vaccine initiation uptake. Yet, the quality and specificity of these relationships remain to be explored. Since community engagement can help identify barriers to vaccination at the local level and thus might lead to sustainable solutions in a manner that a top-down approach cannot achieve [34], measuring these efforts might help to evidence the importance of partnerships in increasing HPV vaccination [35].

### Implications for practice

Our findings underscore the importance of targeted IP strategies in enhancing HPV vaccine uptake. The statistically significant associations between specific state-level activities and vaccine initiation highlight the potential for tailored public health interventions. For instance, increasing transparency through publicly available coverage data and expanding vaccination opportunities in schools and pharmacies may offer practical, scalable solutions to improve HPV vaccination rates. Evaluating the impact of these activities in the context of public health goals can help prioritize resource allocation and guide future interventions.

By developing and disseminating dashboards that present county-level coverage and exemption rates, policymakers can enhance community engagement and inform families about vaccination status in their areas. By providing data on vaccinations, IISs help inform public health actions to improve vaccination rates [36]. This data can help identify potential facilitators and barriers in immunization efforts across geopolitical regions [36]. Such initiatives may ultimately lead to improved vaccination rates and better public health outcomes, underscoring the importance of accessible information in driving vaccination efforts. Implementing these strategies in states with lower vaccine uptake could be instrumental in achieving national public health objectives and reducing the burden of HPV-related cancers.

Due to their accessibility to the general population, making HPV vaccines readily available in community pharmacies could be crucial in increasing HPV vaccination rates. This finding, however, needs to be interpreted with caution as it might be driven by the study design since we used *provider data* for this analysis. Perhaps increasing the number of pharmacists enrolled in the VFC program would not be positively associated with improved vaccination rates if these states had a shortage of enrolled pharmacists. It has been documented that HPV vaccination in the community pharmacy setting has been limited. Previous research conducted in a southern, mostly rural state in the USA found only 15.9% of pharmacies offered the HPV vaccine; only 4.4% had administered at least 1 dose in the past year [37]. Hence, it is important to consider the distribution and quantify these enrolled pharmacists nationwide. It is possible that these pharmacists are in a region within the state already saturated with pharmacists enrolled in the VFC program, whereas other regions might still be lacking. Future density analyses might help elucidate these findings.

Expanding the number of pharmacists enrolled in the VFC program for HPV vaccines coincides with reduced initiation rates among older teenagers. The VFC program aims to provide free vaccines to children whose parents or guardians may not be able to afford them. Consequently, areas that increased pharmacist enrollment in the program may have had low vaccination rates to begin with compared to areas that did not. These regions likely expanded pharmacist enrollment as a strategy to address their already low vaccination rates.

Moreover, adding more pharmacists to the VFC program does not guarantee that more individuals will initiate vaccination. It’s possible that the parents or guardians, or the teenagers themselves, remain unaware of the increased number of pharmacists enrolled in the program. Without effective communication and outreach to inform the community about the availability of vaccines, simply increasing the number of pharmacists may not lead to higher vaccination rates.

The aggregate approach of IP strategies that reach beyond traditional health systems has been discussed prior [38] and studies as a mechanism that may reduce dropout and increase coverage [39]. The opportunity to capture a positive individual impact of vaccination using community strategies also led to implementing strategies other than school-entry policies. Although they have proven to be effective in increasing HPV vaccine uptake in some states in which the mandate has been enacted [27], it may spark hesitancy in different settings.

### Implications for policy

Understanding the specific contributions of these IP activities in HPV vaccine uptake can help shape evidence-based policies and programs that effectively address vaccine hesitancy and barriers to vaccination. Given that IP activities could influence the uptake of the HPV vaccine, this gives insight to public health programs on what approaches may be important for promoting the HPV vaccine. To maximize the impact of these activities that were demonstrated to be significantly associated with higher HPV vaccine initiation rates, it could be beneficial to implement these in states that have not incorporated these activities. However, this could depend on their respective state resources. By adopting approaches proven to be effective in increasing HPV vaccine initiation, public health programs can address existing gaps in vaccine coverage and improve overall population health by ultimately decreasing the burden of HPV-related cancers. Moreover, the associations observed between HPV vaccine initiation and IP activities might be higher prospectively as HPV vaccination continues to be prioritized. This is especially important now due to the post-COVID-19 problem of vaccine uptake for children/adolescents [40], with VFC provider orders for HPV vaccines decreasing during 2020, 2021, and 2022 compared to 2019 [41].

### Strengths and limitations

Results should be considered in the context of limitations. The AIM survey did not include an operational definition for activities, so IP respondents used their perception to define and perform the activity. Therefore, how some activities are carried out could vary between jurisdictions since a program could achieve them in numerous ways. The NIS-Teen survey uses a standard survey methodology to collect data from all studied geographic areas, providing population-based results. Despite this, the NIS-Teen user’s guide cautions that findings using data from the 2019 survey are subject to a few limitations [18]. Telephone surveys are used to gather data, and while efforts are made to adjust for non-responses and households lacking cell phones, there may be some bias [18]. Additionally, the reliance on provider-reported HPV vaccination histories may lead to underestimations of HPV vaccination coverage, as the completeness of these vaccination records cannot be verified [18]. Moreover, although we found that (**E**) states expanding the number of school-located programs entering HPV in IIS and (**N**) states expanding the number of pharmacies entering HPV vaccination data in IIS presented higher initiation rates, the AIM survey did not collect information on how many schools and pharmacies were added in the states that had expansions, thus limiting the ability to quantify and measure the impact of said expansions.

Despite the inherent limitations of missing data, our approach significantly assures the reliability of our findings. This study utilizes a robust methodology that integrates data from the National Immunization Survey for Teens (NIS-Teen) and the AIM Annual Survey, providing a comprehensive examination of HPV vaccine initiation across diverse demographic and geographic contexts. The reliance on provider-reported vaccination histories minimizes potential bias, thereby enhancing the accuracy of the vaccination prevalence estimates. By focusing on both individual-level and state-level variables, the analysis captures a nuanced understanding of the factors influencing HPV vaccine uptake, contributing to the validity of the findings.

Furthermore, the application of multilevel modeling represents a significant strength of this study. This approach effectively accounts for the hierarchical nature of the data, recognizing that vaccination behaviors are shaped by individual characteristics as well as broader state-level factors. The use of a mixed Poisson regression model with random effects not only improves the precision of parameter estimates but also addresses potential clustering within states, providing a more accurate assessment of the association between immunization program activities and HPV vaccine initiation. This methodological rigor offers valuable insights for public health interventions aimed at increasing HPV vaccination rates among adolescents.

## Conclusion

Effective Immunization Program strategies and transparent communication are essential for significantly enhancing HPV vaccine initiation rates among adolescents. Future efforts should concentrate on quantifying these strategies, assessing their implementation in states with lower vaccination rates, and identifying barriers to ensure equitable access to vaccinations. Engaging communities in this process is crucial to tailor interventions to local needs. By prioritizing evidence-based interventions and encouraging policymakers and healthcare providers to take active roles, public health programs can improve overall vaccination coverage, reduce HPV-related health disparities, and ultimately lower the incidence of HPV-related cancers.
